# Antibiotic prescribing patterns in Emergency Department at Regional Hospital in South Africa

**DOI:** 10.4314/ahs.v21i4.19

**Published:** 2021-12

**Authors:** Nahyan Almansoori, Nivisha Parag

**Affiliations:** Emergency medicine division, University of Kwazulu-Natal Republic of South Africa

**Keywords:** Antimicrobial resistance, antibiotic stewardship, emergency department

## Abstract

**Background:**

Antibiotic resistance is a major public health concern. The Emergency department (ED) is the community gate for healthcare where antibiotics are often prescribed. However, there is a paucity of data regarding antibiotic prescription practices in Africa.

**Objectives:**

To describe the use of antibiotics in an ED and level of prescribing adherence to national guidelines.

**Methods:**

Retrospective observational study of antibiotic practice in ED. All patients who presented to ED during the study period and were prescribed an anti-microbial agent were included. Data on demographics, working diagnosis, anti-microbial prescribed, dose, route and prescriber level were used to provide descriptive statistics of these parameters.

**Results:**

We identified 195 (13.4%) patients who received anti-microbial therapy among 1454 charts reviewed. The mean age was 34.8 with male predominance. The most common indication identified was abscess in 37 (30.8%) patients and in general surgical conditions had the highest rate of antimicrobials prescribed at 54.3%. In addition, co-amoxiclav was the most commonly prescribed anti-microbial (72.15%). We found that combination therapy was not common practice in ED, with majority of the patients having received single anti-microbial therapy (87.18%). The appropriateness of antimicrobial prescriptions was (46.2%) and not statistically significant (P = 0.654).

**Conclusion:**

The most commonly prescribed anti-microbial was co amoxiclav and the most common indication was abscess. It was found that antibiotic prescription appropriateness was acceptable when compared to studies conducted in developed countries. However, further research within other hospital departments will add to the study to determine the adherence as an institution rather than the Emergency department alone, as antimicrobial resistance is a major global healthcare problem and impacts patient care throughout the care pathway.

## Introduction

Globally the health community is rapidly approaching the ‘post-antibiotic era’, as evidenced by the existing and increasing identification of multi- and pan- antibiotic resistant infections in hospitals^1^. Albeit a multifaceted problem, antibiotic misuse is the single largest contributing factor for this global health crisis^2^. Annually, it is estimated that more than 19 million sepsis (formerly severe sepsis) cases and 5 million sepsis-related deaths are reported - the majority in low and middle income countries (LMICs)^3^.

The Emergency Department (ED) is often the first healthcare contact point for patients presenting to hospital and ED physicians are tasked with rapid triage and diagnosis of sepsis to enable timeous medical interventions. It has been shown that every hour delay in administering antibiotic therapy results in increased mortality by 7.8% per hour delay after the first hour^4^. Therefore empiric antibiotic use in the ED is a common practice, given that the diagnosis of sepsis is not always easy or specific and microbiological confirmation is not immediately available.

The ED has been identified as a major contributor influencing antimicrobial use and resistance patterns in both ambulatory and inpatient settings. Since EDs are at the interface of the inpatient and outpatient settings, ED practitioners have the unique opportunity to influence antimicrobial stewardship in both locations with important downstream implications^5^. Antibiotics that are incorrectly prescribed contribute drastically to the proliferation of resistant bacteria.

As a result, antimicrobial resistance (AMR) limits therapeutic resources by depleting antibiotic options, especially for hospital-acquired infections. Furthermore, the consequences of AMR are not limited only to reduced antibiotic efficacy, but also increasing costs associated with using agents that are more potent. In addition, intravenous formulations, the use of multiple agents to treat a single infection episode and increasing treatment failure rates contribute to increased patient mortality.

In recognition of AMR being a global health crisis, the United Kingdom (UK) government set a target in 2016 to reduce inappropriate antibiotic prescribing by 2020 to 50%^6^. Better diagnostic coding, more precise prescribing guidelines and a deeper understanding of appropriate long-term uses of antibiotics would allow for identification of further reduction potentials^6^.

Previous research has shown that the treatment indication, choice of agent or duration of antibiotic therapy is incorrect in 30% to 50% of cases presenting to the ED^7^. This was seen particularly in single drug regimens where correct dosing is essential to decrease the development of resistance^8^. When indicated, an antibiotic must be the right choice of agent at the right dose, dosing interval and route, prescribed for the right duration. When not indicated, antibiotics must not prescribed^9^.

A retrospective analysis showed that 9% of all patients who presented to an ED in Denmark received antibiotics. Additionally, up to 65% of prescriptions were inappropriate in some conditions e.g. Urinary Tract Infection (UTI)^10^. Similarly, research conducted by Zeng et al., in a Chinese pediatric Emergency department, showed that 62% (n= 311) of 500 of patients received antibiotics. In the majority of these cases, the use of antibiotics was identified to be inappropriate^11^. Over 80% of children with either a wheezy chest or bronchitis received atibiotics. Antibiotic use for children with an Upper Respiratory Tract Infection (URTI) or tonsillitis in this study was greater than the 20% maximum recommended by the European Surveillance of Antimicrobial Consumption (ESAC).

A systematic review and meta-analysis of community-acquired Blood Stream Infections (BSI) in Africa found that only 13 % of adult patients presenting with fever had BSI with the most common causes being Salmonella typhi, non- Typhoid Salmonella (NTS), Brucella species and Streptococcus Pneumonia^12^. Patients in low-income countries (LICs) were more likely to present to hospital with infection than those in High-income countries (HICs). South Africa is defined by the World Bank as an upper-middle income country it is likely that the incidence of infection in general and BSI in particular, would lie in the region between HICs and LICs^13^. Mortality for severe sepsis is between 15% and 30% in high-income countries, whereas it is 50% or higher in low-income countries^14^.

In patients presenting to the Emergency department with a chief complaint of diarrhea, a South African study conducted by Kudsk-Iversen et al., showed that 60% of these patients received antibiotics, yet only 34% had an organism identified on stool microscopy or culture. In all cases, the final diagnosis was listed as acute gastroenteritis without further specification and antibiotic use according to national guidelines appeared inconsistent^15^.

The decision-making process in the ED setting concerning prescribing antibiotics needs to be both rapid and appropriate. Furthermore, adherence to local and updated guidelines regarding prescribing practices (antibiotic choice, dose, dosing interval and duration) would help in reducing the problem of AMR and lessen injudicious use of antibiotics. Antimicrobial stewardship programs may be beneficial by focusing on prescribing guidelines for common conditions seen among ED patients. Moreover, creation of local guideline pocketbooks may serve to improve prescribing practices and meet the Core Elements of Outpatient Stewardship recommended by the Centers for Disease Control and Prevention16. Initiatives promoting antibiotic stewardship has been shown to be effective in South Africa when examined in internal medicine wards at a public hospital in Cape Town where antibiotic prescriptions were reduced to 20% and costs reduction by 35% 17.

There is limited available data describing antibiotic usage in emergency departments locally. It is thus difficult to measure the compliance to the antimicrobial stewardship program guidelines. This study aimed to describe the antimicrobial prescribing practices at a busy regional ED in KwaZulu-Natal and to identify any factors amenable to practice change to help in reducing the burden of antimicrobial resistance.

## Methods

### Study design

This was a retrospective chart review conducted in the Emergency Department at Edendale Hospital. The study was conducted from 1st March to 31^st^ March 2018.

### Study setting

Edendale Hospital (EDH) is a 900-bed healthcare facility located in the uMgungundlovu District in Kwa-Zulu-Natal. It provides regional-level health care to 1.4 million people. The emergency department manages an average of 25000 to 30000 patients per annum and has a 30% hospital admission rate. Consultant emergency physicians are typically present from 08h00 to 16h00 on weekdays and weekends with telephonic assistance outside these hours and on site assistance if needed. The patient population predominantly comprises of adults with acute medical, surgical and trauma emergencies, and pediatric trauma. Paediatric medical and surgical conditions are excluded from this department and attended to by a 24 hour operational pediatric outpatient department.

### Data collection

Patients' data were extracted from the ED admission records, and included the retrieval of discharge records and case files from medical registry. Data collected was entered into a Microsoft excel spreadsheet generated for this purpose – variables captured included patient age, gender, diagnosis, antibiotic received, dose, route, interval and prescriber level. Patient identity was not extracted to maintain confidentiality of medical records. The study included all patients who presented to the ED in the defined period of study, including walk-in, ambulance arrivals and inter-facility referrals. Furthermore, there were no exclusion criteria other than missing records where data extraction was not possible.

### Sample size

Using the Kish Leslie formula (1965): Where n= z2 x p (1-p)/ d2

n = required sample size;

z = standard error of the mean which corresponds to 95% confidence level (standard value of 1.96)

p= percentage of population exposed

d = margin of error at 5% (standard value of 0.05)

### Statistical analysis

Data was entered into SPSS version 25 (Statistical Packages for the Social Sciences) for analysis. A p value <0.05 considered as statistically significant. A descriptive statistical analysis of the data (means, standard deviations, ranges, frequencies and percentages) was done to address the objectives of the study.

To evaluate the prescriber adherence to guidelines, The Standard Treatment Guidelines and Essential Drug List (EDL) for South Africa Adult hospital Level Edition 2015 was used as a reference.

### Ethical consideration

The study was approved by University of Kwa-Zulu-Natal Health Biomedical Research Ethics Committee (BREC) of the faculty of Health Sciences of the University of KwaZulu-Natal (Reference number: BE504/18) and was registered in the KZN Department of Health database.

## Results

Overall, 2377 patients presented to the ED during the study period of one month. Of these, 923 were excluded due to missing records or missing data relevant to the study (n = 918), patients being transferred to another facility (TB hospital, n=3) and two deaths were reported. Thus, 1454 of 2377 (61.2%) of patients seen were included in the data analysis. 1072 females (45%) and 1305 males (55%) attended the ED during the study period. Patient ages ranged from 7 months to 104 years with a mean age of 34.88 (SD = 188.09) and median age of 31 (IQR=23). The ratio of patients who received antibiotics at the ED was 195/1454 (13.41%) during the period under review. The majority were males, 115 (59%) vs 80 (41%) females who received antimicrobials. More than half of the patients (73%, n = 143) given anti-microbial therapy were within the age groups 20 – 59 years old (Table 1).

**Table 1:** Age distribution and female to male ratio for patients received anti-microbial therapy

Age group	Distribution	Female	Male	Total
**1**	0.1 – 5 years	1	5	6
**2**	6 – 10 years	4	8	12
**3**	11 – 19 years	6	8	14
**4**	20 – 29 years	22	36	58
**5**	30 – 39 years	15	20	35
**6**	40 – 49 years	11	18	29
**7**	50 – 59 years	10	11	21
**8**	60 – 69 years	8	5	13
**9**	70 – 104 years	3	4	7
**Total**		80	115	195

Indications for antimicrobial prescriptions were mostly surgical reasons (54.36%), followed by medical (21.54%), trauma (11.79%), orthopedics (9.74%), and the least being gynecological and urological cases (2.56%). The most common diagnosis identified was abscess collection in 37 (18.97%) cases and the least common were Post-Exposure Prophylaxis (PEP) and abdominal distention of any cause (0.51%). Lower respiratory tract infection was diagnosed in 14 patients (7.17%) and found to be the most common medical indication for anti-microbial prescription in ED. (Table 2)

**Table 2:** Indications for antibiotics

Diagnosis	Frequency	Percentage
Abscess	37	18.97%
Appendicitis	18	9.2%
Cellulitis	17	8.71
Tonsillitis	7	3.58%
Septic Wound	6	3.07%
UTI	5	2.56%
Acute Cholecystitis / Cholangitis	4	2.05
UGIB	2	1.02%
Gangrene	2	1.02%
Sepsis / infection	21	10.7%
LRTI	14	7.17%
Post TB Bronchiectasis	4	2.05%
AGE	4	2.05%
Hypoglycemia	3	1.53%
Meningitis	3	1.53%
DKA secondary to infection	3	1.53%
Asthma / COPD	2	1.02%
Injury	13	6.66%
GSW	8	4.1%
MVA	4	2.05%
Fracture	9	4.6%
Septic arthritis	4	2.05%
PID	3	1.53%
PEP	1	0.51%
Abdominal Distension	1	0.51%
**Total**	195	100%

**Graph 1:**
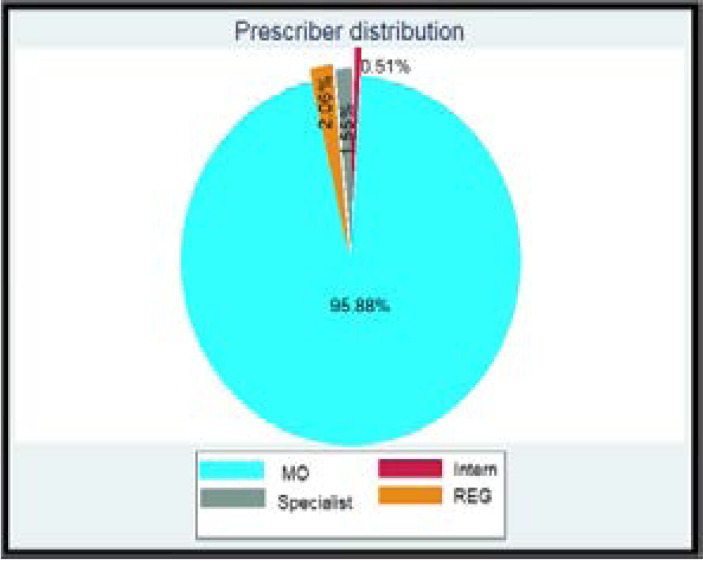
Prescriber distribution at ED

Regarding prescriber l evels of antimicrobial in ED, 96% of agents were prescribed by medical officers and 3% prescribed by Registrars and specialists. Intern doctors (in training post undergraduate studies) only prescribed anti-microbial agents to two patients. (Graph 1)

**Graph 2:**
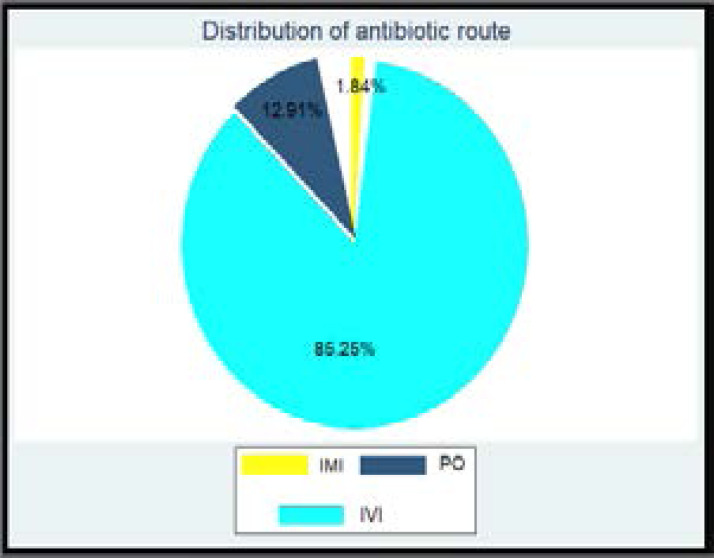
Administration Route

A total of 217 antimicrobial prescriptions were identified. The intravenous route was found to be the most commonly used route n = 185 (85.25%) followed by oral route n=28 (12.91) % and least intramuscular route n = 4 (1.84%) (Graph 2)

The majority of patients (n = 170, 87.18%) received a single anti-microbial agent. A total of 22 patients (11.28%) received dual therapy and three patients received triple therapy (1.54%). The combination of co-amoxiclav and azithromycin was the most common combination therapy prescribed to 15 patients (7.69%). Co-amoxiclav was prescribed for 138 patients as a single agent (70.77%) and was a part of combination therapy in 20 patients (Table 3).

**Table 3:** Anti-microbial agents prescribed at E.D

Antibiotics	Freq.	Percent
Ampicillin	1	0.51
Ampicillin + Ceftriaxone	1	0.51
Augmentin	138	70.77
Augmentin + Azithromycin	15	7.69
Augmentin + Azithromycin +ceftriaxone	1	0.51
Augmentin + Metronidazole	2	1.03
Augmentin + Metronidazole + Fluconazole	1	0.51
Augmentin + acyclovir + fluconazole	1	0.51
Azithromycin	2	1.03
Cefazolin	7	3.59
Ceftriaxone	13	6.67
Ceftriaxone + Azithromycin	2	1.03
Ciprofloxacillin	1	0.51
Cloxacillin	6	3.08
Cloxacillin + floxacillin 1 g QID	1	0.51
Floxacillin	1	0.51
Metronidazole	1	0.51
Tazocin + clindamycin	1	0.51

**Total**	195	100

Prescriptions were compared to national guidelines using the EDL 2015 Adult hospital level. To determine if the correct choice of antibiotics were prescribed to the patients, a ‘Yes’ and ‘No’ was assigned to each prescription, and 46.2% (n=89) were in accordance with the guidelines, while 48.2% (n = 94) were not compliant with the guidelines. This result showed a similar incidence of correct vs incorrect prescribing practice which was not significant (p =0.654). There were 5.6% (n = 11) of prescriptions for antimicrobials with no clear indications for its use (Table 4).

**Table 4:** Correct choice at time of prescription in comparison to EDL 2015 hospital level

Correct choice	Female	Male	p-value
**Yes**	35	55	0.654
**No**	40	54	
**No clear indication**	5	6	1.000
**Total**	80	115	

The correct dose was prescribed in 92.8% (n = 181) of cases while the incorrect dose was identified in 7.2% (n = 14). Table 5.

**Table 5:** Correct dosage at time of prescription in comparison to EDL 2015 hospital level

Correct dose	Female	Male	p-value
**Yes**	74	107	1.000
**No**	6	8	
**Total**	80	115	

## Discussion

This study describes the antimicrobial usage in a busy emergency department at a regional government hospital in KwaZulu-Natal during a one month period. It shows essential understanding into the current practice of antimicrobial prescribing in the ED. Furthermore, the data examined the characteristics of all included patients who received an antimicrobial, rather than limiting data collection to a single disease condition. This information can provide multiple insights including the overall recognition of frequency of antimicrobials being prescribed in the ED, the most common conditions for these prescriptions and antimicrobials with the highest usage. The appropriateness of antimicrobial prescriptions was assessed only on the provisional diagnosis given by treating physician in the ED, which was based on clinical examination and available investigations excluding cultures. This method provided a practical approach to the decision of empiric antimicrobial prescribing habits in the ED. This differs from many previous studies assessing antimicrobial appropriateness, where appropriateness has been defined based on culture and susceptibility results.

Culture results are often unavailable to the ED clinician at the time of antimicrobial prescribing. Moreover, cultures are not routinely done for each antimicrobial prescription as most of patients who received antimicrobials are not systemically ill. Therefore, measuring appropriateness of antimicrobial prescription by conducting retrospective study based on blood culture might not always be valuable in an emergency setting. Additionally, blood cultures prior to antimicrobial prescriptions were not routinely performed in this ED and turn-around times for blood culture results are 5–7 days.

A recent retrospective study conducted in Australia showed that 1 in 8 (13.4%) presentations to the ED will receive antimicrobials^18^. This is comparable to the results of this study, where 1 in 7 (13.6%) patients had been prescribed antimicrobial agent, with a suggestion of adherence to rational prescribing practices in the ED. In contrast, a prospective study conducted in Chinese pediatric emergency department showed that 1 in 1.6 presentations will be prescribed antimicrobials^11^.

Regarding the appropriateness of prescription, the Australian study showed that 1 out 3 (33.3%) of prescriptions were either sub-optimal or inadequate. The most common reasons for antimicrobial prescriptions to be inappropriate were the broadness of a spectrum and there was a lack of indication or unnecessary overlap of spectrum or antimicrobial choices. The rate of suboptimal prescribing in this study was 1 in 1.8 (55.6%) prescriptions that deviated from the EDL guidelines, comparative to a study conducted at a pediatric emergency department in Spain^19^. This finding can partly be explained by prescribing decisions that are either based on local institutional guidelines or consultations with other specialist departments. This identifies a need for further examination of prescribing practices in the ED.

Additionally, the Australian study showed that the most common indication for antimicrobial prescriptions were skin infections of any type (17%) and can be compared to the diagnosis of abscesses receiving the most antimicrobials in this study (18.9%). Antimicrobials for respiratory tract infections were used with a frequency of 15.7% compared to 7.1% in this study. Further studies in the United States (US) showed anti-microbial prescription inappropriateness rates of up to 40%^20^. A recently published cross sectional study in US showed inappropriate prescription level of 23.3%. Furthermore, it showed that 28.5% of prescriptions had no clear indication^21^. This is in contrast with this study, where the indication was not clear in only 5% of the cases, albeit a smaller sample size.

Addressing most commonly prescribed antimicrobials, this study shows that beta-lactam agents, more specifically penicillins were prescribed the most, with co-amoxiclav being the most frequently used agent (70.77%). In an African setting, a study conducted within a tertiary level hospital in Nigeria also showed that co-amoxiclav was the most commonly prescribed agent (43%). However, regarding combination therapy co-amoxiclav and metronidazole were dominant in dual therapy unlike our study findings, which shows co-amoxiclav and azithromycin as the commonest combination therapy^22^. In contrast, a recent retrospective study from the United States conducted in ED showed that penicillin prescription was significantly lower (22.3%)^23^.

## Limitations

Several limitations were identified in this study. Most importantly, this is a retrospective study with basic risks of error as it used data from an available database and from non- electronically recorded data. Data entered might be incomplete, imprecise or invalid, resulting in information bias. We acknowledge that incomplete data might influence the findings of this study. Furthermore, a sample representative of the patient population of interest was not optimal as the study included only acute and emergency presentations and excluded walk in medical adult patients and all pediatric medical patients. Data collators were not blinded to the study objectives and hypothesis. A possible approach to compensate for this shortcoming in the future is to emphasize transparency in documentation and possibly collect data prospectively. An improved medical record system will help minimize missing patients' records.

## Conclusion

This study describes the current anti-microbial use and practices in a busy public hospital in South Africa. It showed that approximately half of all prescriptions were inappropriate. Therefore, it might add value to curbing the AMR problem. This study is a mandatory starting point for a root cause analysis in the ED to assist in identifying the prescribing gaps and conducting an assessment specifically to improve the adherence to antibiotic stewardship programs.

## Data Availability

Medical Records at Edendale regional Hospital with local hospital and department of health permission.
